# Prevalence and associated factors of reading spectacle coverage among adults aged 35 years and above living in Debre Berhan Town, North Shewa, Ethiopia, 2023

**DOI:** 10.3389/fopht.2025.1496499

**Published:** 2025-04-16

**Authors:** Matiyas Mamo Bekele, Melkamu Temeselew Tegegn, Nebiyat Feleke Adimassu, Abel Sinshaw Assem, Tarekegn CheklieZeleke, Abebizuhan Zigale Bayabil, Getenet Shumet Birhan, Abebech Fikade Shumye

**Affiliations:** Department of Optometry, College of Medicine and Health Sciences, Comprehensive Specialized Hospital, University of Gondar, Gondar, Ethiopia

**Keywords:** spectacle, near vision spectacle coverage, adults, Debre Berhan, North Shewa, Ethiopia

## Abstract

**Introduction:**

Near vision impairment can be addressed through several methods, including spectacles, contact lenses, miotic drugs, and refractive surgery. Of these options, spectacles are the most commonly used, affordable, and accessible solution. Reading spectacle coverage is an important indicator of admittance and eye care service utilization. Additionally, it serves as a valuable tool for monitoring progress toward achieving universal eye health coverage worldwide.

**Objective:**

The aim of this study was to assess the prevalence and associated factors of reading spectacle coverage among adults aged 35 years and above living in Debre Berhan town, North Shewa, Ethiopia.

**Methods:**

A community-based cross-sectional study involving 808 adults was conducted in Debre Berhan town from May 8 to June 8, 2023, utilizing a multistage sampling technique. Data were gathered using a pre-tested, structured questionnaire administered by interviewers. The information was collected through Kobo Collect version 2021.4.4 and subsequently exported to Stata version 14 for processing and analysis. A binary logistic regression analysis was performed to identify the factors associated with reading spectacle coverage. Variables with a p-value of less than 0.05 in the multivariable binary logistic regression were deemed statistically significant.

**Results:**

A total of 780 participants took part in the study, resulting in a response rate of 96.53%. The average age of the participants was 49.58 ± 9.31 years. The proportion of reading spectacle coverage was 32.69% (95% CI: 28.82, 36.31). Factors positively associated with reading spectacle coverage included higher educational status (AOR = 3.10, 95% CI: 1.59, 6.05), awareness of near vision problems (AOR = 3.24, 95% CI: 2.08, 5.05), a history of eye examination (AOR = 3.16, 95% CI: 1.58, 6.55), experiencing difficulties with near vision (AOR = 2.56, 95% CI: 1.26, 5.21), and adding plus lens power used ≥2.50D (AOR = 1.26, 95% CI: 1.13, 3.16).

**Conclusion:**

The study found that the proportion of reading spectacle coverage was low. A higher level of education, history of awareness of near vision problems, history of difficulties in near vision, history of eye examination, and high adding lens power used were significantly associated with reading spectacle coverage.

## Introduction

Presbyopia is an age-related change in the eye that leads to difficulty focusing on nearby objects. It is most common in individuals between the ages of 40 and 45 and is more prevalent in women (1). The primary cause of near visual impairment in adults is presbyopia, which is clinically observed as a decrease in the amplitude of accommodation due to changes in the lens’s growth and flexibility (2, 3). Near Visual Impairment (NVI) is defined as difficulty in performing near tasks at a distance of 40 centimeters, with near visual acuity of 6/12 (N8) or lower (4).

In 2005, approximately 1.04 billion people worldwide were affected by presbyopia. This figure is expected to rise to 1.4 billion by 2020 and to 1.8 billion by 2050 (5). Moreover, the prevalence of near visual impairment in East Africa, including Ethiopia, varies between 61.7% and 85.4% (6). Refractive errors and presbyopia can be effectively corrected with spectacles, which are also the most cost-effective solution (7, 8). However, coverage for reading spectacles varies significantly; for example, it was just 0.7% in a study conducted in rural China, while it reached 99.5% in a study conducted in the United States (2, 9).

Presbyopia can significantly affect an individual’s quality of life and productivity, regardless of their literacy level (10, 11). It leads to a decline in near vision, which can result in economic losses for those whose livelihoods depend on good near vision, especially if the condition is left uncorrected (1, 12). Research indicates that presbyopia compromises visual function, making it difficult to perform essential tasks such as harvesting, writing, cooking, and sorting. This condition also impacts daily activities, near-vision tasks, and social interactions (13–15).

Presbyopia can be addressed through various methods, including glasses, contact lenses, miotic medications, and refractive surgery. Among these options, spectacles are the most commonly used, as they are the most affordable and accessible method of correction (12). Addressing presbyopia is crucial for achieving sustainable development goals that promote health and well-being for everyone (16).

The World Health Organization (WHO) suggests prioritizing services for presbyopia, a common age-related vision issue. Their guidelines state that a population should be deemed a high priority for these services if fewer than one-third of individuals affected have access to near vision correction (17). If one to two-thirds of the population uses spectacles, they are classified as a moderate priority. In contrast, a population is regarded as low priority if more than two-thirds already have spectacles (17). To improve accessibility, alternative strategies are necessary, as many optical suppliers and hospitals charge high prices for eyewear. A widely used approach to make presbyopic spectacles more accessible involves community distribution programs. These programs may train individuals as “vision guardians” or “village entrepreneurs” who can distribute eyewear to those in need (18). This approach is sustainable as it depends on community engagement and integrates with existing local health worker networks or village distributors (19).

The availability of reading glasses coverage is a key indicator for assessing access to and utilization of eye care services. It also aids in tracking advancements towards achieving universal eye health coverage worldwide (20). This metric is used to evaluate the effectiveness of health systems in providing high-quality eye care and achieving the desired health outcomes (21).

The study population consisted of elderly individuals, as they tend to have a higher prevalence of near-visual impairment (6, 22). While previous studies in Ethiopia and other regions have primarily focused on distance visual impairment, there is limited information on reading spectacle coverage throughout the country, and even less regarding the specific study area of Debre Berhan town in North Shewa. Therefore, the aim of this study is to assess the prevalence and associated factors of reading spectacle coverage among adults aged 35 years and above living in Debre Berhan town, North Shewa, Ethiopia.

## Materials and methods

### Study design, period and area

A community-based cross-sectional study was carried out in Debre Berhan town, located in North Shewa, Ethiopia, from May 8 to June 8, 2023. The town is 130 kilometers from Addis Ababa, the capital of Ethiopia, and 688 kilometers from Bahir Dar, the capital of the Amhara National Regional State. Debre Berhan has a total population of 88,375, which includes 39,961 males and 48,414 females. Of this population, 56,914 individuals are adults aged 35 and older (23). The town is divided into nine administrative regions, known locally as kebeles. It is served by a tertiary eye care center with ophthalmologists, optometrists, and ophthalmic nurses, catering to over 3 million people in the surrounding area. Additionally, there is a private eye clinic in the town, along with more than three optical workshops.

### Study population and eligible criteria

All adults aged 35 years and older who had resided in Debre Berhan for at least six months during the data collection period were eligible to take part in the study. However, individuals who were not responding to the questionnaire due to serious illness or mental impairment were excluded from participation.

### Sample size determination

The sample size was calculated using the single proportion population formula $\text{n}=\frac{{\left(\text{Z}\alpha {/}_{2}\right)}^{2}\text{x P}(1-\text{P})}{{\text{d}}^{2}}$ where n is the sample size, Z is the z statistic value at a 95% confidence level (1.96), P is the expected proportion of reading spectacle coverage (28.42%) based on a previous study conducted in Finote Selam, Ethiopia (24), and d is the margin of error (5%). This calculation resulted in a sample size of 489. After accounting for a design effect of 1.5 and a 10% non-response rate, the final planned sample size increased to 808.

### Sampling technique and procedures

Debre Berhan town consists of nine kebeles. To ensure a representative sample, a multi-stage sampling procedure was implemented. Initially, four kebeles were randomly selected from the nine. The sample size was then allocated proportionally according to the population of each chosen kebele. A systematic random sampling technique was used to select households, with a fixed interval of 14, which was calculated by dividing the total number of households in the selected kebeles (10,519) by the sample size (808). To determine the first household, a random number between 1 and 14 was generated, and every 14^th^ household thereafter was included in the sample. If multiple eligible adults aged 35 years or older were present in a selected household, participants were chosen using a lottery method. If no eligible individuals were found during data collection, the household was revisited twice. If there were still no eligible individuals meeting the criteria, the survey included an immediate neighboring household.

### Operational definitions

Reading spectacle coverage (%): was calculated as 100 times the number of participants currently using reading spectacles, divided by the total number of participants who either have current near vision correction or do not use any near vision correction (24).
Met need: A participant who can see the N6 line or better with their current near correction at 40 cm (25).
Under met need: A participant who could not see N6 or worse with their current near correction improved to N6 or better with the new correction (3).
Unmet need: A participant could not see the N6 line without correction but could see it clearly at a distance of 40 cm with correction (3).
Awareness: Was used to describe individuals who recognize age-related near vision problems, commonly referred to as presbyopia (26).
Near visual impairment (NVI is defined as having near visual acuity worse than 6/12, according to the updated International Classification of Diseases (ICD-11). NVI is categorized based on severity as “mild” when the presenting near visual acuity (PNVA) from 6/12 to 6/18 (equivalent to worse than N8 to N10), “moderate” when the PNVA is from 6/18 to 6/60 (equivalent to worse than N10 to N18), and “severe” when the PNVA is worse than 6/60 (equivalent to worse than N18) (3, 9, 27).
Previous eye examination: If the individual had a previous eye visit within the past 2 years (28).

### Data collection tool and procedure

The data collection tool was adapted from previous similar studies (24, 26). The questionnaire included demographic data, information on vision and eye care services, behavioral factors, and medical and clinical conditions. Six trained optometrists gathered the data through face-to-face interviews and physical examinations. They used a pen-torch light for examining the anterior segment and direct ophthalmoscopes for the posterior segment. The questionnaire was initially developed in English by reviewing relevant literature and considering current clinical practices, then translated into Amharic and back-translated into English by language experts to ensure accuracy and reliability. Before the actual data collection, the Amharic version of the questionnaire was pretested on 5% of the sample size to assess reliability and quality, with corrections made based on feedback received. To measure distance and near visual acuity, both distance Snellen and reduced Snellen acuity charts were utilized. Subjects with a distance visual acuity of ≤ 6/9 underwent refraction using a streak retinoscope. Near visual acuity (NVA) was evaluated binocularly after achieving optimal distance correction, using reduced Snellen acuity charts at a distance of 40 cm. Additionally, near refraction was conducted binocularly on top of distance correction for all subjects with near visual acuity less than N6, using spherical plus lenses until the individual could read at least N6 or better.

### Data processing and analysis

The data was entered into Kobo Collect version 2021.4.4 and subsequently exported to Stata version 14 for analysis. Descriptive statistics, including frequency and percentage, were utilized to summarize the data. To assess the multicollinearity of variables, the variance inflation factor (VIF) and tolerance tests were performed. Binary logistic regression was applied to identify factors associated with reading spectacle coverage. Variables with a p-value of less than 0.2 in the bivariable analysis were included in the multivariable regression analysis. The goodness of fit for the model was evaluated using the Hosmer–Lemeshow test. In the multivariable logistic regression analysis, variables with a p-value of less than 0.05 were considered statistically significant at the 95% confidence interval.

## Results

### Socio-demographic characteristics of the study participants

A total of 780 participants were involved in this study, resulting in a response rate of 96.53%. The average age of the participants was 49.58 years (SD ± 9.31). Of the 780 participants, 556 (71.28%) were male, 629 (80.64%) were married, and 401 (51.41%) had attained higher education (college level or above) (**Table 1**).

**Table 1:** Socio-demographic characteristics of study participants in Debre Berhan town, North Shewa, Ethiopia, 2023 (n = 780).

Variables	Categories	Frequency	Percentage
Sex	Female	224	28.72
	Male	556	71.28
Marital status	Not married	93	11.92
	Married	629	80.64
	Divorced	37	4.74
	Widowed	21	2.69
Educational status	Unable to read and write	98	12.56
	Able to read and write	162	20.77
	Primary school	71	9.10
	Secondary School	119	15.26
	College and above	330	42.31
Occupational status	Merchant	196	25.13
	Government employed	346	44.36
	Housewife	88	11.28
	Private employed	118	15.13
	Unemployed	32	4.10
Income per month (ETB)			
	<6000	301	38.58
	6001-7349	225	28.84
	7350-8000	142	18.20
	>8000	112	14.35

### Eye care service utilization and awareness among participants

Two hundred fifty-five participants (32.69%) reported being aware of near-visual impairment. Additionally, 522 participants (66.92%) were knowledgeable about refraction sites, and 370 participants had undergone eye check-ups within the past year (**Table 2**).

**Table 2:** Vision and eye care service information-related factors of study participants in Debre Berhan town, North Shewa, Ethiopia, 2023 (n = 780).

Variables	Categories	Frequency	Percent
Awareness about presbyopia	Yes	255	32.69
	No	525	67.30
History of eye examination	Yes	255	32.69
	No	525	67.31
Difficulties in near vision activities	Yes	522	66.92
	No	258	33.08
Awareness about refraction services delivered	Yes	522	66.92
	No	258	33.08
Family history of spectacle	Yes	367	47.05
	No	413	52.95
Reason for not wearing of reading spectacle (n=780)	High cost	419	53.71
	Unavailability	63	8.07
	Fear of stigma	75	9.61
	Spectacle weakens eye	68	8.71
	Discomfort from weight	35	4.48
	Lost/broken	30	3.84
	Unwillingness	90	11.53
History of diabetes	Yes	62	7.95
	No	718	92.05
Level of near visual impairment	Mild	359	68.85
	Moderate	111	21.28
	Severe	52	9.96
History of Hypertension	Yes	13	1.67
	No	767	98.33

### Proportion of reading spectacle coverage among study participants

The current study revealed that the proportion of reading spectacle coverage was 32.69% (95% CI: 28.82, 36.31). Among the participants, 522 (66.92%) were identified as having near vision impairment (NVI). Of these, 474 individuals (90.90%) had correctable presbyopia, while 48 cases (9.10%) were classified as non-correctable conditions. More than half of the participants, 359 (68.85%), experienced mild NVI, followed by 111 participants (21.28%) with moderate NVI. Furthermore, the “met need” for presbyopia care was reported for 279 individuals (35.77%; 95% CI = 32.48–41.84).

### Factors associated with reading spectacle coverage

In the bi-variable binary logistic regression analysis, factors such as being female, older age, higher education level, occupational status, a history of eye examinations, difficulties with near vision activities, awareness of near vision problems, the need for plus lens power greater than 2.50D, a history of cataract surgery, and a family history of spectacle use were found to be significantly associated with reading spectacle coverage. However, in the multivariable binary logistic regression analysis, significant associations with reading spectacle coverage were observed for higher education level, a history of eye examinations, difficulties with near vision activities, awareness of near vision problems, and the requirement for plus lens power greater than 2.50D.

This study found that participants with a higher level of education (college and above) were 3.10 times more likely to have reading spectacle coverage compared to those who were illiterate (AOR = 3.10, 95% CI: 1.59, 6.05).

Participants with a positive history of awareness of near vision problems had odds of reading spectacle coverage that were 3.24 times higher than those without such awareness (AOR = 3.24, 95% CI: 2.08, 5.05). Additionally, the odds of reading spectacle coverage for participants with a positive history of eye examinations were 3.16 times greater compared to their counterparts (AOR = 3.16, 95% CI: 1.58, 6.55).

Furthermore, participants who reported difficulties with near vision had 2.56 times greater odds of having reading spectacle coverage compared to those who did not have such difficulties. (AOR = 2.56, 95% CI: 1.26, 5.21). Participants who used adding plus lens power of higher than 2.50D had 1.26 times better odds of reading spectacle coverage compared to participants who used adding plus lens power between 0.75-2.00D (AOR = 1.26, 95% CI: 1.13, 3.16) (**Table 3**).

**Table 3:** Factors associated with reading spectacle coverage among adults in Debre Berhan Town, North Shewa, Ethiopia, 2023 (n =780).

Variables	Reading Spectacle Coverage				
	Yes	No	COR (95% CI)	AOR (95% CI)	P-value
**Age (years)**			1.05 (0.27-3.98)	0.43 (0.08-2.18)	0.315
Sex					
Female	158	66	0.94 (0.59-1.16)	1.27 (0.81-1.97)	0.288
Male	399	157			
Educational status					
Unable to read and write	23	75	1.00	1.00	
Able to read and write	37	125	0.96 (0.53-1.74)	0.90 (0.46-1.78)	0.781
Primary school	13	58	0.73 (0.34-1.56)	0.80 (0.34-1.88)	0.618
Secondary School	47	72	2.12 (1.17-3.85)	2.81 (1.37-5.79)	0.005
College and above	135	195	2.25 (1.34-3.78)	3.10 (1.59-6.05)	0.001
Occupational status					
Merchant	63	133	0.93 (0.64-1.36)	1.50 (0.92-2.44)	0.101
Government employed	116	230	1.00	1.00	
Housewife	31	57	1.07 (0.65-1.76)	1.93 (1.02-3.81)	0.041
Private employed	33	85	0.76 (0.48-1.21)	0.77 (0.44-1.34)	0.357
Unemployed	12	20	1.18 (0.56-2.39)	1.27 (0.49-3.25)	0.617
Awareness of near vision problems					
Yes	211	44	2.78 (1.92-4.03)	3.24 (2.08-5.05)	<0.0001
No	332	193	1.00		
History of eye examination					
Yes	74	181	0.31 (0.22-0.43)	3.16 (1.58-6.55)	<0.0001
No	296	229	1.00	1.00	
Difficulties in near vision activities					
Yes	348	174	0.94 (0.79-1.50)	2.56 (1.26-5.21)	0.018
No	175	83	1.00	1.00	
Adding plus lens power used (D)					
0.75-2.00	164	381	1.00	1.00	
2.25-2.50	52	73	1.65 (1.10-2.46)	1.51 (0.91-2.50)	0.106
>2.50	39	71	1.27 (0.82-1.96)	1.26 (1.13-3.16)	0.039
History of cataract surgery					
Yes	58	197	0.58 (0.42-1.26)	0.76 (0.48-1.21)	0.258
No	197	394	1.00	1.00	
Family history of spectacle use					
Yes	137	230	1.48 (1.10-2.01)	1.37 (0.92-2.04)	0.114
No	118	295	1.00	1.00	

COR, Crude Odd Ratio, AOR, Adjusted Odd Ratio; CI, Confidence Interval, and D-Diopter.

## Discussion

In this study, the proportion of reading spectacle coverage was 32.69% (95% CI: 28.82, 36.31). The finding of this was lower than the previous studies conducted in India (35.1%) (7), and China (44.12%) (29). This discrepancy may be due to the variation of socio-demographic characteristics, history of eye examination, and study population. For example, studies conducted in India and China focused on elderly populations, which may lead to an overestimation of reading spectacle coverage, as the demand for such spectacles tends to increase with age.

Conversely, the result of this study is higher than the studies done in Finote Selam (28.42%) (24), Nigeria (3.4%) (30), and Zanzibar (17.6%) (31). The discrepancy may be attributed to variations in the study settings and populations. In Nigeria, the majority of participants were female, while in the current study, most participants were male, particularly among spectacle users compared to females (26, 29).

Participants with a higher level of education (college and above) were 3.10 times more likely to have reading spectacle coverage compared to those who were illiterate. The findings of this study align with those from previous research conducted in Finote Selam (24, 24), Ethiopia (24), and China (29). This relationship may be explained by the fact that individuals with higher levels of education are generally more aware of the importance of vision care and the potential risks associated with uncorrected vision issues. They are more likely to understand the benefits of wearing near-vision spectacles and seek appropriate eye care services. Additionally, educated individuals typically have better access to information about vision care through formal education, media, and healthcare services. As a result, they may be more adept at understanding and navigating the healthcare system to obtain vision correction services. Therefore, higher levels of education can facilitate access to near-vision spectacles by enhancing awareness, improving access to resources, increasing health literacy, providing better employment opportunities, and expanding social networks. These factors contribute to a greater likelihood of seeking and obtaining vision correction services among educated individuals compared to those who are illiterate.

Participants with a positive history of awareness of near vision problems had odds of reading spectacle coverage that were 3.24 times higher than those without such awareness. This finding aligns with earlier studies done in Finote Selam (24), and Hawassa, Ethiopia (26). This relationship might be due to the fact that having a positive history of awareness of near vision problems is a key factor in ensuring reading spectacles coverage. This awareness encourages early intervention, proactive healthcare-seeking behavior, preventive actions, and treatment compliance. Additionally, it contributes to improved quality of life, better performance in educational and occupational settings, and enhances overall social and psychological well-being.

The odds of reading spectacle coverage for participants with a positive history of eye examinations were 3.16 times greater compared to their counterparts. The finding of this study was consistent with the previous studies conducted in Finote Selam (24), and Hawassa, Ethiopia (26). The probable reason for this relation might be due to positive history of eye examination. A positive history of eye examinations is essential for reading spectacle coverage. It enables early detection and accurate prescription of glasses, facilitates treatment of underlying conditions, and allows for regular monitoring of near vision. Furthermore, it promotes preventive care, provides education and counseling on near vision care, aids in navigating healthcare options, and maximizes the use of health insurance for eye care services.

The odds of reading spectacle coverage among participants with a positive history of difficulties in near vision were 2.56 times higher as compared to those who had no history of difficulties in near vision. This finding is agreed with the studies conducted in Hawassa, Ethiopia (26) and China (32). The reason for this positive relation might be due to positive history of near vision difficulty. This awareness can lead to several outcomes: it encourages individuals to recognize their need for vision correction, motivates them to seek professional eye care, promotes compliance with prescribed treatments, enhances quality of life and performance, prevents further eye strain, and increases awareness about the importance of regular eye care and vision monitoring.

Participants who used adding plus lens power of higher than 2.50D had 1.26 times better odds of reading spectacle coverage compared to participants who used adding plus lens power between 0.75-2.00D. This finding aligns with earlier studies done in Finote Selam, Ethiopia (24) and China (32). The likely reason for this association is that individuals who require higher near add often face significant challenges with near tasks, especially when they are uncorrected. As a result, they are more motivated to seek out glasses or spectacles to enhance their productivity and improve their quality of life.

### The strength and limitations of this study

The strength of this study lies in its provision of updated information on reading spectacle coverage and the use of a sufficiently large sample size, which increases the study’s power. However, there are some limitations. As a cross-sectional study, it can only demonstrate the time-based relationship between predictors and reading spectacle coverage, without establishing a definitive cause-and-effect relationship. Furthermore, individuals living on the streets were not included, as the sampling was restricted to households.

## Conclusion

This study found that the prevalence of reading spectacle coverage was low. Factors significantly associated with reading spectacle coverage included a higher level of education, awareness of near vision problems, a history of difficulties with near vision, previous eye examinations, and the use of high adding lens power.

## Data Availability

The original contributions presented in the study are included in the article/supplementary material. Further inquiries can be directed to the corresponding author.
